# 2627. Natural History of RSV Infection in Outpatient Episodes Among Children in the United States using Two EHR Data Sources by COVID-19 Era

**DOI:** 10.1093/ofid/ofad500.2240

**Published:** 2023-11-27

**Authors:** Diana Garofalo, Sima Toussi, Joshua T Swan, Maria Kudela, Padmalatha Reddy, Emily Webber, Sally R Omidvar, Stephen E Schachterle, Suzanne Landi, Anindita Banerjee, Margaret Tawadrous, Niki Alami, Scott P Kelly

**Affiliations:** Pfizer, New York, New York; Pfizer, New York, New York; Pfizer, New York, New York; Pfizer, New York, New York; Pfizer, New York, New York; Truveta, Beaverton, Oregon; Truveta, Beaverton, Oregon; Pfizer Inc., Brooklyn, New York; Pfizer, New York, New York; Pfizer, New York, New York; Pfizer, Inc, Groton, Connecticut; Pfizer, New York, New York; Pfizer, New York, New York

## Abstract

**Background:**

Respiratory syncytial virus (RSV) is ubiquitous and infects almost all children during the first two years of life; infants (<1 year) and children with high-risk conditions (preterm, congenital heart disease, chronic lung disease, immunocompromised) are at an increased risk for severe infection and hospitalization. Most patients are managed at home, yet data are sparse on the natural history of RSV infection in non-hospitalized populations. To address this gap, the natural history of RSV infection in children (< 2 years) initially managed in the outpatient setting was examined in two US data sources, by COVID-19 era.

**Methods:**

In Optum and Truveta, both US-based, electronic health record databases, infection episodes among children (< 2 years) with a positive RSV laboratory test (real-time PCR or antigen) in the ambulatory setting were identified prior to (Optum: 2016-2019, Truveta: 2017-2019), or during (Optum: 2020-2021, Truveta: 2020-2022) the COVID-19 pandemic. Episodes were excluded if immediately hospitalized (within 24 hours of index), and multiple infections per patient were allowed if >28 days apart. First occurrence of all-cause hospitalization was assessed 14 and 28 days after index.

**Results:**

During the pre-COVID-19 era, mean age ranged from 8.6-8.7 months, while during the COVID-19 era, mean age was slightly higher at 9.7-9.8 months (Table 1). Infants < 1 month were most commonly hospitalized after an initial outpatient visit, compared to older infants (Table 2). In 28 days of follow-up, the hospitalization proportion among children < 2 years ranged from 4.8-5.6% pre-COVID-19 and 3.6-5.4% during the COVID-19 pandemic (Table 2), similar to values using 14 days of follow-up. Risk was higher in Truveta than Optum; hospitalization in neonates (< 1 month) increased in the COVID-19 era compared to pre-COVID-19, while the opposite was true for children < 2 years.

**Figure:**
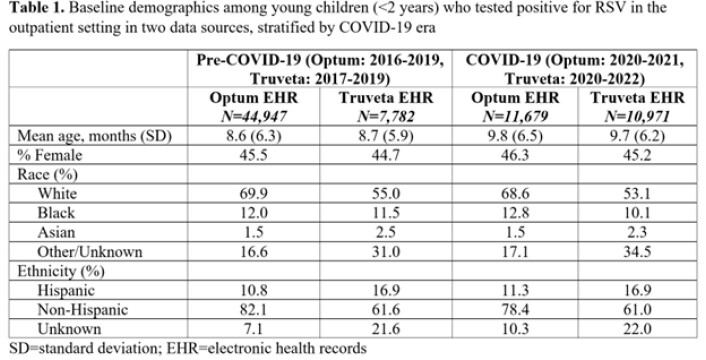

**Figure:**
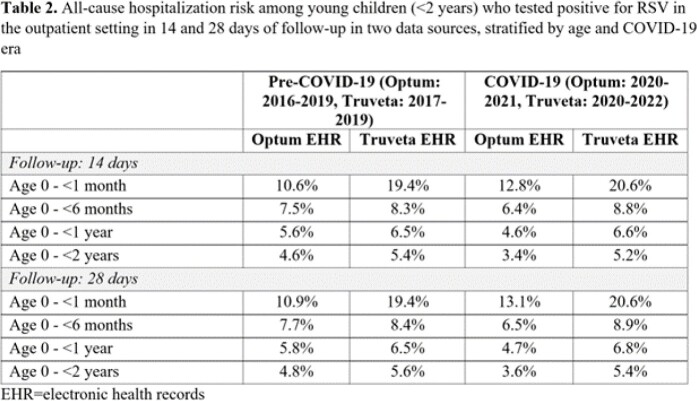

**Conclusion:**

Most RSV outcomes occurred within 14 days of a positive test. Despite healthcare disruptions of the COVID-19 pandemic, an appreciable proportion of children were hospitalized after RSV infection diagnosed in the outpatient setting, especially among infants < 1 year. Future work will investigate sources of heterogeneity in results across data sources.

**Disclosures:**

**Diana Garofalo, PhD MPH**, Pfizer: Stocks/Bonds **Sima Toussi, MD**, Pfizer: Stocks/Bonds **Joshua T. Swan, PharmD, MPH, BCPS, FCCM**, CareDx: Grant/Research Support|Genentech: Grant/Research Support|Grifols Share Services North America: Grant/Research Support|Heron Therapeutics: Grant/Research Support|Kedrion Biopharma: Advisor/Consultant|Kedrion Biopharma: Grant/Research Support|Pacira Pharmaceuticals: Grant/Research Support|Pfizer: Grant/Research Support|Pfizer: Employee|Pfizer: Stocks/Bonds|VigiLanz Corporation: Grant/Research Support **Maria Kudela, PhD**, Pfizer: Employee|Pfizer: Stocks/Bonds **Padmalatha Reddy, Ph.D.**, Pfizer: Stocks/Bonds **Stephen E. Schachterle, Pfizer Inc.**, Pfizer Inc.: Full time employee|Pfizer Inc.: Ownership Interest|Pfizer Inc.: Stocks/Bonds **Suzanne Landi, PhD**, Pfizer, Inc: Employment|Pfizer, Inc: Stocks/Bonds **Anindita Banerjee, Phd**, Pfizer: Employee and stockholder **Margaret Tawadrous, MD, MS**, Pfizer: Full time employee|Pfizer: Full-time employee|Pfizer: Full-time employee|Pfizer: Full -time employee|Pfizer: Ownership Interest|Pfizer: Ownership Interest|Pfizer: Ownership Interest|Pfizer: Ownership Interest|Pfizer: Stocks/Bonds|Pfizer: Stocks/Bonds|Pfizer: Stocks/Bonds|Pfizer: Stocks/Bonds **Niki Alami, MD**, Pfizer: Employee|Pfizer: Stocks/Bonds **Scott P. Kelly, PhD**, Pfizer: Employee|Pfizer: Stocks/Bonds

